# Phenotypic, Anatomical, and Diel Variation in Sugar Concentration Linked to Cell Wall Invertases in Common Bean Pod Racemes under Water Restriction

**DOI:** 10.3390/plants11131622

**Published:** 2022-06-21

**Authors:** Karla Chavez Mendoza, Cecilia Beatriz Peña-Valdivia, Martha Hernández Rodríguez, Monserrat Vázquez Sánchez, Norma Cecilia Morales Elías, José Cruz Jiménez Galindo, Antonio García Esteva, Daniel Padilla Chacón

**Affiliations:** 1 Programa de Posgrado en Botánica, Colegio de Postgraduados, Carretera México-Texcoco, km 36.5, Montecillo 56230, Mexico; karla.chavez@colpos.mx (K.C.M.); cecilia@colpos.mx (C.B.P.-V.); vazquez.monserrat@colpos.mx (M.V.S.); morales.norma@colpos.mx (N.C.M.E.); esteva@colpos.mx (A.G.E.); 2 Postgrado en Recursos Genéticos y Productividad-Genética, Colegio de Postgraduados, Carretera México-Texcoco, km 36.5, Montecillo 56230, Mexico; hernandez.martha@colpos.mx; 3 Campo Experimental Sierra de Chihuahua-INIFAP, Ciudad Cuauhtémoc 31500, Mexico; cruz2477@yahoo.com.mx; 4 CONACYT-Programa de Posgrado en Botánica, Colegio de Postgraduados, Carretera México-Texcoco, km 36.5, Montecillo 56230, Mexico

**Keywords:** *Phaseolus vulgaris*, water restriction, raceme fruit, pod wall, sucrose, cell wall invertase

## Abstract

The common bean (*Phaseolus vulgaris* L.) pod wall is essential for seed formation and to protect seeds. To address the effect of water restriction on sugar metabolism in fruits differing in sink strength under light–dark cycles, we used plants of cv. OTI at 100% field capacity (FC) and at 50% FC over 10 days at the beginning of pod filling. Water restriction intensified the symptoms of leaf senescence. However, pods maintained a green color for several days longer than leaves did. In addition, the functionality of pods of the same raceme was anatomically demonstrated, and no differences were observed between water regimes. The glucose and starch concentrations were lower than those of sucrose, independent of pod wall size. Remarkably, the fructose concentration decreased only under water restriction. The cell wall invertase activity was twofold higher in the walls of small pods than in those of large ones in both water regimes; similar differences were not evident for cytosolic or vacuolar invertase. Using bioinformatics tools, six sequences of invertase genes were identified in the *P. vulgaris* genome. The PvINVCW4 protein sequence contains substitutions for conserved residues in the sucrose-binding site, while qPCR showed that transcript levels were induced in the walls of small pods under stress. The findings support a promising strategy for addressing sink strength under water restriction.

## 1. Introduction

Pod set and filling in common bean (*Phaseolus vulgaris* L.) occur by the transportation of photoassimilates from leaves to fruits [1]. Qualitative studies on temporal and spatial aspects of flowering and fruiting have indicated that flower number and pod set vary with environmental conditions and gene pool [2,3]. It is well known that in the phloem, drought induces sugar limitation and leads to fruit and seed abortion [4,5], to the detriment of seed yield [6]. This effect strongly depends on carbon partitioning mechanisms that must adapt to energy demands and modify the genetic architecture of photosynthate allocation for multiple sinks [1]. In addition to all the negative effects induced by drought, the evidence indicates that although the seed number is significantly reduced during drought, seed filling is maintained at levels similar to those of seeds developing under optimal conditions [1,7]. This indicates that the accumulation of seed weight remains linear under low water availability, even when the source:sink ratios change. The pod wall plays a crucial role in regulating carbon partitioning during seed filling under stress and under conditions without stress [1,7,8]. However, when leaf photosynthesis is potentially limited, carbohydrate partitioning is shifted from pod walls toward seeds [9,10]. Several lines of evidence have demonstrated that the legume pod wall can photosynthesize and makes an important contribution to seed yield in species, including alfalfa (*Medicago sativa*) [11], chickpea (*Cicer arietinum*) [12], soybean (*Glycine max*) [13], and lentil (*Lens culinaris* L.) [14]. Thus, the physiological responses to drought in the pod wall may be due to the adjustment of metabolism to maintain homeostasis and seed filling. Further studies have demonstrated that the pod wall accumulates starch during the early stages of pod development [15]. For instance, [16] reported that pods accumulate starch in response to drought stress. However, the functions of some important starch-degrading enzymes during drought stress remain to be investigated. Experimental evidence indicates that changes in the levels of sugars, including fructose, glucose, and sucrose, in pods are more noticeable during the early stages of pod development in response to drought stress [17]. Sucrose degradation is a well-known factor in responses to abiotic stresses when phloem-unloaded sucrose must be degraded into glucose and fructose or their derivatives by invertase (INV, EC 3.2.1.26) or sucrose synthase (SUS, EC 2.4.1.13) [18]. The functional characterization of invertases (INVs) and their regulatory mechanisms have been identified in crops under abiotic stress [19,20]. In particular, cell wall invertase (CWIN) is a key enzyme in sucrose metabolism that catalyzes the breakdown of sucrose into glucose and fructose, which serve to prevent programmed cell death (PCD) by scavenging reactive oxygen species (ROS) [21,22]. These findings indicate that the role CWIN activity could be vital for fruit set under abiotic stress due to higher rate of sucrose import into young fruit [23]. In this regard, some isoforms could modify the control of sucrose metabolism in a highly specific manner. Recently, the use of bioinformatic analysis provided the identification of 18 transcription factors as putative regulators of the expression of *AtCWIN2* and *AtCWIN4* that are predominantly expressed in Arabidopsis reproductive organs [24].

Here, we hypothesized that the water restriction differentially alters the distribution and accumulation of photoassimilates between pod wall when the foliage is severely affected. The identification of CWIN gene expression in reproductive organs of common bean represents a significant advancement to understand the mechanism in sucrose partitioning and metabolism. In this study, we demonstrated the possible links between cell wall invertase activity and encoding *PvINVCW4* gene identified by a genome-wide analysis within the latest genome annotation. The results provided evidence for a possible physiological of pod wall in the context of sucrose metabolic under water restriction stress.

## 2. Results

### 2.1. Substrate Moisture

The soil moisture level at 100% FC showed variable daily losses, and they were recovered by applying a 0.24–0.43 mL water g^−1^ substrate. At 50% FC, soil water moisture decreased quickly after 4 days (Figure S2). To avoid excessive stress, water was replenished daily to 50% FC (0.22 mL water g^−1^) for 10 days.

### 2.2. Analysis of Senescence by Color Segmentation on Visible RGB Images

**Figure 1:**
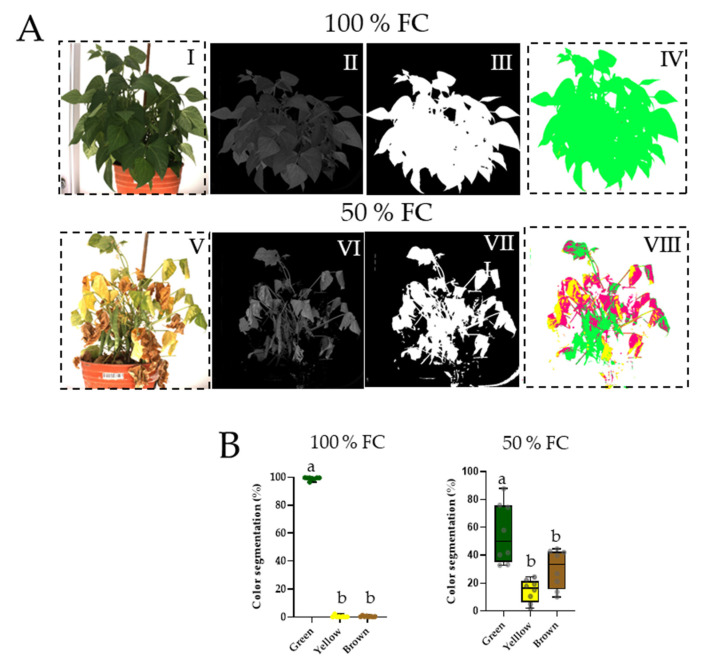
(**A**) Color segmentation of RGB images of common bean plants of cv. OTI in soil with 100% field capacity (FC) and after 10 d at 50% FC. The images were obtained with a Scanalyzer PL and analyzed by LemnaGrid Software. Foreground and background separation of the images and resulting binary images (**I**–**III**,**V**–**VII**). Objects were separated according to color classification (**IV**–**VIII**). (**B**) Percentage (± S.E.) of color in green (healthy), yellow (senescent), and brown (necrotic) plants, *n* = 5. Boxes marked with a different letter were significantly different (*p* < 0.05) by Student’s *t* test.

Plants at 100% FC maintained 100% green color throughout the 10-d evaluation (Figure 1A,B). In contrast, water restriction significantly modified the green–yellow–brown color components between 40–60% (Figure 1A). The leaves of stressed plants showed strong symptoms of senescence, and significant necrosis was even observed. Images of stressed plants clearly show that the fruits were permanently green in comparison with the yellowish and necrotic leaves (Figure 1B).

### 2.3. Morphological and Anatomical Analysis

**Figure 2:**
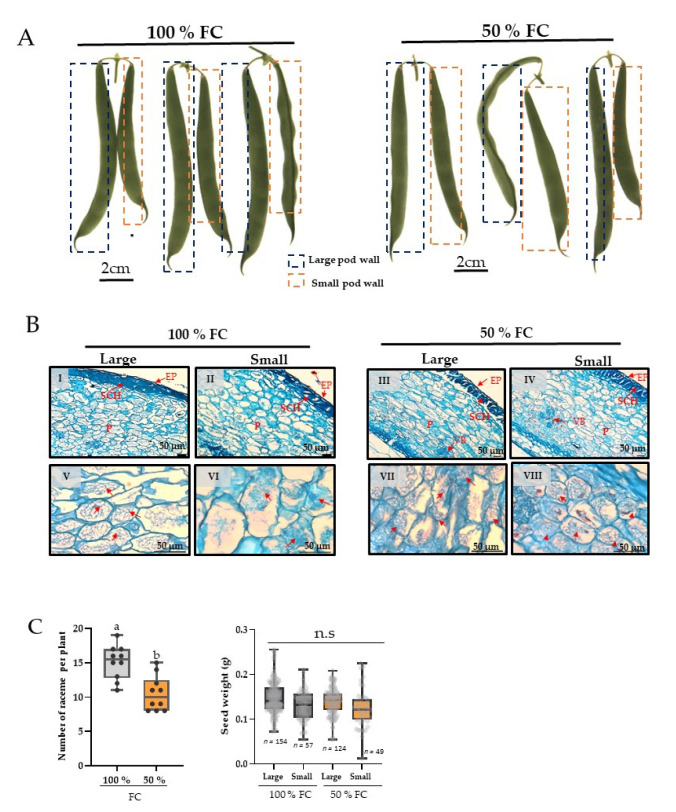
(**A**) Examples of pod racemes with large and small fruits from plants at 100% field capacity (FC) and under water restriction (50% FC) for 10 d. (**B**) Anatomical characteristics of walls of large and small pods developed in common bean plants with soil at 100% field capacity (FC) and after 10 days at 50% FC. (**I**–**IV**): pericarp cross-sections (10× objetive); ((**V**–**VIII**); light micrographs (40× objetive) showing starch granules in parenchyma (P) (red arrows). Epidermis (EP), sclereid hypodermis (SCH), vascular bundles (VB). (**C**) Racemes per plant harvested after 10 d; *n* = 10. Left graph. Seed weight (±S.E.) distribution in large and small pods. Right graph, from plants at 100% FC (gray) and 50% FC (orange); *n* = 5. Boxes marked with a different letter were significantly different (*p* < 0.05) when analyzed by Student’s *t* test. n.s = not significant.

The racemes of 26–29 DDA were harvested after 10 days of stress and images were acquired (Figure 2A). The racemes’ shape and morphology strongly indicated that all racemes with large and small green fruits were able to maintain metabolic activity, i.e., photosynthesis, compared with other plant tissues. In addition, the anatomical analysis showed that the external layer (exocarp) and sclereid hypodermis were similar between both treatments (Figure 2BI–BIV). In addition, the cells of the parenchyma (mesocarp) showed no differences in the number or shape of the cells. Moreover, starch grains were present in the pod wall of the large and small pods of both treatments, but they were only in the cells of parenchyma (Figure 2BV–BVIII).

Seed and raceme production at 50% FC fell 30%, while seed production decreased from 15–20% (Figure 2C). Remarkably, no significant differences in individual seed weight were found at 50% FC with respect to the control, indicating that despite water restriction, the fruits were able to complete grain development (Figure 2C).

### 2.4. Diel Variation in Soluble Sugars and Starch in the Pod Wall

**Figure 3:**
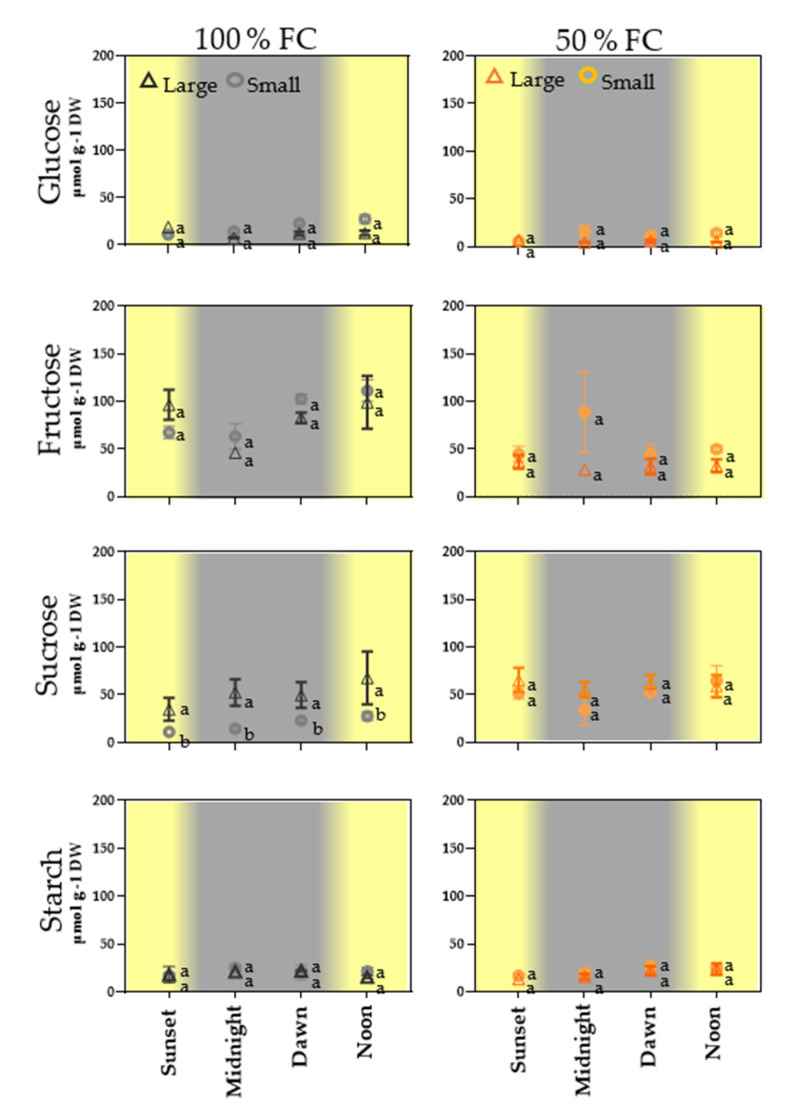
Concentrations ± SE of glucose, fructose, sucrose, and starch in pericarps of large (triangle) and small (circle) pods of racemes of common bean plants at 100% field capacity (FC) over 10 days. Racemes were sampled at sunset (6:00 p.m.), midnight (12:00 p.m.), sunrise (6:00 a.m.), and noon (12:00 p.m.), *n* = 4. The same letters on the boxes indicate similarity between treatments (Student’s *t* test, *p* < 0.05).

The diel range of glucose concentration (10–18 µmol g^−1^) in the pod walls was the lowest among the soluble sugars in both treatments. In contrast, the fructose concentrations showed peaks as high as 99 µmol g^−1^ in the walls of large and small pods at 100% FC during daylight. Fructose concentrations were not so high at 50% FC in pod walls of either size, with the concentrations of this monosaccharide reduced by half. Slight diel changes in sucrose concentration were observed in the walls of large pods at 100% FC. Under water restriction, sucrose concentrations were maintained without changes (55–65 µmol g^−1^). Changes in starch concentrations (16–25 µmol g^−1^) were proportionally lower than those of sucrose (Figure 3).

### 2.5. Diel Variation in Invertase Activity in the Pod Wall

**Figure 4:**
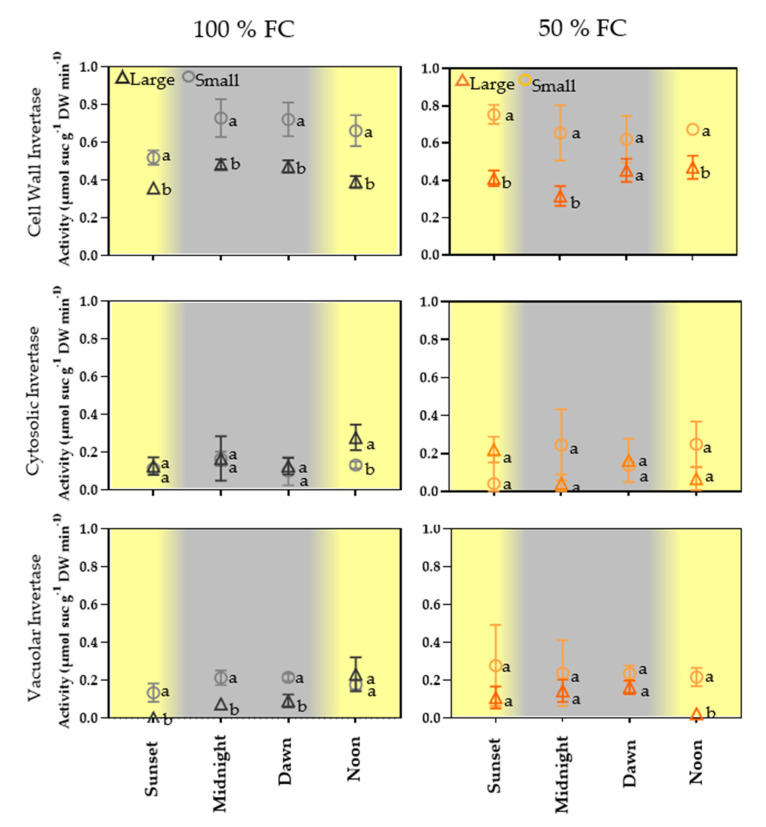
Activities ± SE of cell wall invertase, cytosolic invertase, and vacuole invertase in pericarp from pod sets (large and small) at 100% FC and over 10 d at 50% FC. The pod sets were sampled at sunset (6:00 p.m.), midnight (12:00 a.m.), dawn (6:00 a.m.), and noon (12:00 p.m.); *n* = 4. The same letters on the boxes indicate similarity between treatments (Student’s *t* test, *p* < 0.05).

Diel light/dark variation in CWIN activity was significantly higher (0.67–0.72 µmol suc g^−1^ DW min ^−1^) in the walls of small pods than in those of large ones (0.31–0.48 µmol suc g^−1^ DW min ^−1^) in both treatments. The difference represents 35–40% of CWIN activity (Figure 4). In contrast, CIN and VIN activity was lower (0.15–25 µmol suc g^−1^ DW min ^−1^) than that of VIN; however, the activity of VIN was slightly higher in the walls of small pods at 50% FC (Figure 4).

### 2.6. INVCW Isoforms within the P. vulgaris Genome

A search was conducted for all possible CWIN-like genes from the list of 38 INVCW gene sequences (Table S2), and these CWIN-like genes were then curated. The Table S2 shows the IDs and chromosome locations. All gene sequences were grouped into the glycoside hydrolase family GH32. Through alignment of all gene sequences, the genome of *P. vulgaris* was unloaded in Phytozone V.13. Extensive analysis via iterative BLASTs at the protein level was performed; we determined the percentage similarity values for the 38 INVCW gene sequences (Table S3).

From this analysis, six INVCW sequences were selected using a threshold similarity > 78% and experimentally studied (Table S4). Thus, we propose a standardized gene nomenclature for INVCW isozyme entries, and through ProtParam software (https://web.expasy.org/protparam/, accessed on 20 February 2022), we obtained characteristics such as the ORF lengths, the numbers of amino acids, the isoelectric points (pI), molecular weights, and signal peptides, which were predicted (Table 1). As shown, the nucleotide sequence lengths ranged from 1524 to 1926 bps, deduced protein sequence lengths varied from 575 to 652 amino acids, and the corresponding pIs were predicted to be between 5.05 and 9.82.

The organization of exon/intron of the *PvINVCW* genes are illustrated in Figure S3. All *PvINVCW* genes showed the presence of exons, *PvINVCW1, PvINVCW2*, and *PvINVCW3* genes contained 6–7 exons, while *PvINVCW4, PvINVCW5*, and *PvINVCW6* contained 5–6 exons. 

### 2.7. Analysis of Conserved Domains in the INVCW Sequences

**Figure 5:**
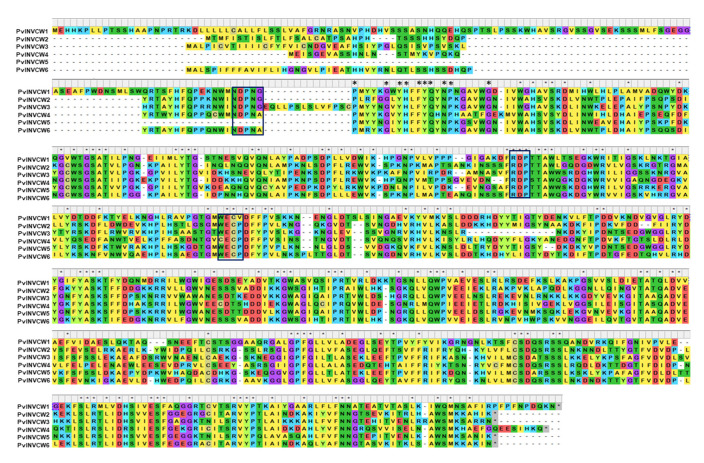
Alignment of deduced amino acid sequences of invertases of *Phaseolus vulgaris.* Strongly conserved residues are indicated above the alignment with asterisks. Potential glycosylation sites are underlined. The β-fructosidase motif, DPN motif, and cysteine catalytic site are boxed in blue.

The alignment of six invertase sequences indicated 141 conserved amino acids, shown as blue boxes in Figure 5. The three conserved motifs NDPNG (β-fructosidase motif), RDP, and WECP, which perform a crucial function in the hydrolysis of sucrose into glucose and fructose, were found in PvINVCW2 and PvINVCW3. Irregular substitution in the NDPNG domain of an A residue for a single G residue was detected in the PvINVCW4 and PvINVCW6 sequences. Similarly, the WECP domain showed substitution of an aliphatic W residue with a C residue in the PvINVCW4 sequences, while in the PVINVCW1 sequence, a P residue was replaced by V (Figure 5).

### 2.8. Phylogenetic Analysis of PvINVs

To understand the phylogenetic relationships among the INVCWs in *P. vulgaris*, a phylogenetic tree was constructed. In this case, the conserved sequences of 15 members of the INV family in dicots and 25 in monocots, as well as one sequence from the bacterium *Bacillus subtilis* subsp. as an outgroup, from the https://www.ncbi.nlm.nih.gov, accessed on 5 February 2022) database were used. Accession numbers for all sequences used in the analysis are in Table S1.

**Figure 6:**
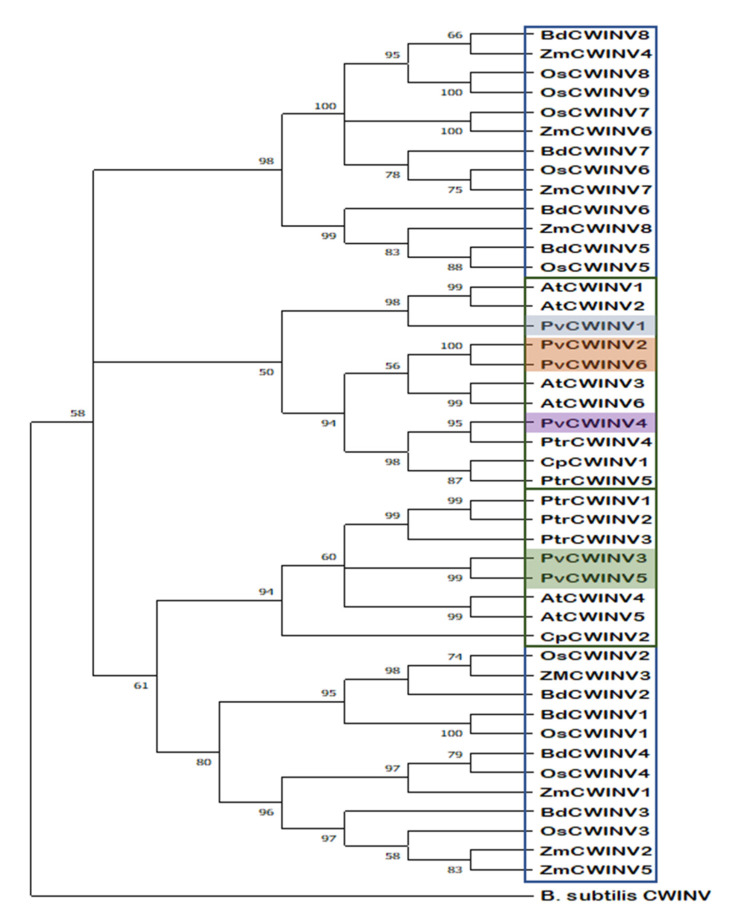
Phylogenetic tree constructed by the NJ method using the MEGA5.2.2 program based on protein sequences of *Phaseolus vulgaris* and invertases from monocotyledonous (Monocot) and dicotyledonous (Dicot) plants and including cell wall invertases of the bacterium *B. subtilis* as an out group. Accession numbers are in Table 1.

All plant invertases that are catalytically active share the common feature of being relatively large proteins with >500 aa, compared to the median size of ~363 aa for plant proteins [25]. Our phylogenetic study covering both dicot and monocot species confirmed that the INV family comprises many members within a species and that they are divided into two main groups, one containing sequences classified as cell wall invertases in monocots and the other in dicots with the distant bacterial sequence, reflecting independent evolutionary origin (Figure 6).

This indicated that the PvINVCWs could be divided into three groups: PvINVCW1 has a close relationship with PvINVCW2 and PvINVCW6, while PvINVCW3 is closest to PvINVCW5. PvINVCW4 was clustered into the subgroup closest to PvINVCW2 and PvINVCW6.

The *PvINVCW* genes were distributed within five out of eleven common bean chromosomes. An extensive analysis at the protein level allowed us to propose a standardized gene nomenclature of all *P. vulgaris* isozymes (Table 1). The genes were grouped into different categories based on the structural features (Figure 6) and the phylogenetic tree (Figure 6).

### 2.9. Analysis of the Expression of Two P. vulgaris Invertases

**Figure 7:**
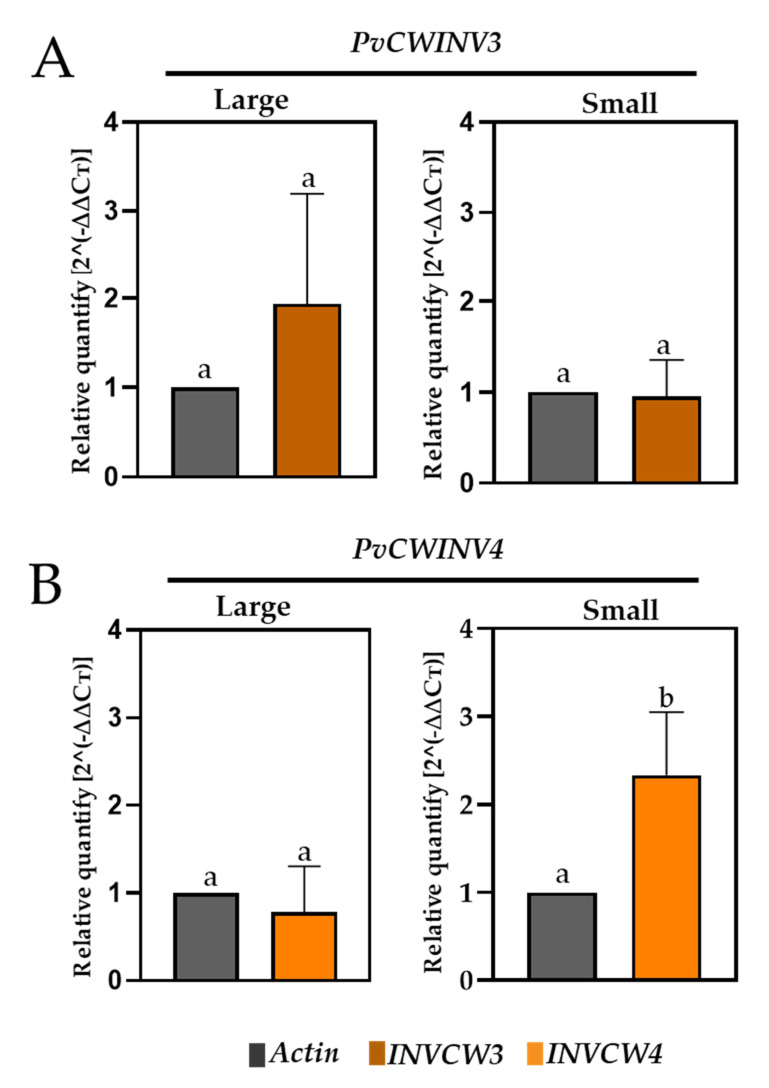
Profiles of qRT–PCR expression ± SE of the *Phaseolus vulgaris* invertase genes (**A**) *PvINVCW3* and (**B**) *PvINVCW4* in tissues of large and small pods and developmental stages at 100% FC (field capacity) and after 10 d at 50% FC. The pod sets were harvested at noon (12:00 p.m.), *n* = 3. The same letters on bars indicate similarity between treatments (Student’s *t* test, *p* < 0.05).

To increase data precision on the expression levels of INVCW genes, we selected the *PvINVCW3* and *PvINVCW4* genes to carry out qRT–PCR based on their high-percentage sequence similarity values (Table S4). In the samples from walls of large pods, expression of *PvINVCW3* and *PvINVCW4* was not significantly different than at 100% FC (Figure 7). In the walls of small pods, *PvINVCW3* gene expression was not different from that in plants at 100% FC. In contrast, *PvINVCW4* gene expression was approximately 2-fold higher than that in walls of small pods at 100% FC (Figure 7).

## 3. Discussion

As much as 80% of the CO_2_ assimilated during photosynthesis is channeled into the synthesis of sucrose [26]. Under heat and drought, sugar limitation is a well-known factor leading to the abortion of fruit and seed [27]. In the common bean, plants initiate many more reproductive structures than can be carried to full seed maturity [28,29]. Pods can be developed in any raceme along the plant [30]. Recent evidence indicates that under water restriction, the carbon allocation in the pod wall changes over time, and ^14^C labelling shows that under water restriction, the seeds increase ^14^C label accumulation with fruit aging and decrease ^14^C accumulation with increasing moisture level [17].

In the present study, we evaluated the effect of water restriction on raceme fruits with different numbers of seeds (Figure 2A). First, we demonstrated that despite strong symptoms of senescence, racemes remained green (Figure 1). This allows us to suggest that pods probably maintain active photosynthetic machinery. In this regard, various studies have recognized the green color permanence in plants, which is known as “Stay Green”, an agronomic character [31]. In common bean, it has been demonstrated that the green color permanence in pods varies among cultivars [32]. To confirm whether pod greenness is related to cellular structural integrity, the anatomy was analyzed in cross-sections of long and small pods of the plants with and without water restriction (Figure 2B). Pod walls did not show alterations in the epidermis (exocarp), hypodermis, or internal (mesocarp) or external parenchyma (endocarp) (Figure 2(BI–BIV)), or in the starch granules (Figure 2(BV–BVIII)). Altogether, these results indicate that even if plants are under water restriction, their fruits maintain cellular integrity similar to those at 100% FC. These observations are consistent with findings on the pods of alfalfa [33], chickpea [34], and some species of *Brassica* [35]. Additionally, it has been shown that the pod provides carbon to the seeds even when growth conditions are adverse; it is a selection criterion for bean cultivars under water restriction conditions [8].

In addition, biomass distribution in pod racemes was evaluated, as was seed yield per plant, in both treatments. The stress generated significant pod abscission (Figure 2C), and the differences in fruit size and number of seeds per pod were affected by water restriction (5–6 seeds per pod to 3–4 seeds per pod) (Figure 2A). Studies with ^14^C have revealed that, under moderate and severe water restriction, fruits in intermediate developmental stages show significant differences in sucrose and starch concentrations with respect to the initial (elongation) and advanced (mature) stages [17]. Our results confirm that between 26 and 29 DAA, common bean pods show active carbon metabolism despite the absence of photosynthetic activity in leaves. The present study suggests that the pod walls are metabolically active structures in racemes with large and small pods. Hence, pod anatomy showed that the cellular integrity was maintained similarly in pod walls despite 10 days of water restriction (Figure 2B). Regarding the weights of the seeds, the results showed that water restriction reduced the fruit number per plant, but individual seed weight was not affected (Figure 2C). This indicates that the seeds complete development despite the stress and the limited, or totally suspended, assimilation of photoassimilates from the leaves. This is consistent with findings of the study that evaluated the genic base of seed formation, which demonstrated that in crops, seed size is a genetically marked trait [36]. In another approach, Ref. [1] evaluated the role of pods in the determination of seed yield under drought and found that the number of seeds per pod was also correlated with the pod harvest index (PHI), suggesting that sink strength due to the seed number is higher than sink strength based on seed size.

Additionally, we explored the effect of the day–night cycle on the levels of soluble sugars and starch in pod walls of large and small fruits (Figure 3). We found that glucose concentrations remained low in walls of both pod sizes regardless of moisture level or time of day. In contrast, the fructose concentration in the pod walls at 100% FC varied between conditions with natural light and darkness. Walls of large and small pods accumulated less fructose at the end of the day and throughout the night, with maximum accumulation occurring at noon (Figure 3). In contrast to 100% FC, water restriction kept fructose concentrations in pods at low levels (Figure 3). These results indicate that the glucose and fructose used in the pods are metabolically different. It is well known that in plants, hexoses (glucose and fructose) are generated by invertase activity. The elevated levels of fructose in bean pod walls were consistent with those documented in response to moisture deficit stress [17] and during the process of chloroplast formation during seed development [37].

Elucidating the metabolic and signaling roles of these sugars can be complex, since they are rapidly metabolized and are substrates for the synthesis of other molecules. However, fructokinase, which catalyzes the same reaction as hexokinase (HXK), is involved in plant growth modulation and the use of fructose as a regulator of 1,6-fructokinase [38]. The findings of the current study have not ruled out the possibility that fructose is involved in fruit growth regulation, which generates new questions. The significant diel changes in sucrose levels in walls of long and small pods (Figure 3) suggest that under water restriction stress, sucrose accumulates via discharge from the phloem or is synthesized from triose phosphate produced by photosynthesis in the pod walls. Sucrose is indispensable to the development of reproductive structures such as flowers and seeds [4,17,39]. For example, in flowers of cotton (especially), sucrose is the major nutrient and energy source [40]. In maize, sucrose applied to the stem has been found to restore ovary abortion in plants subjected to drought [41]. Walls of long and small pods did not show significant differences in starch concentration between treatments or during the light–dark cycle (Figure 3). These results are consistent with those obtained by [15], in which pods of common bean in the early stages of development showed lower starch concentration than did those in the advanced developmental stage. In contrast, Ref. [16] reported that starch levels in the pod wall increased in response to drought in a drought-tolerant cultivar, showing that the starch concentration varies among cultivars. However, further investigation is needed to address the issue of how the pod wall regulates sugar export products resulting from starch degradation, such as soluble sugars and maltose [42].

To gain additional insight into the sucrose:hexose ratio in pod walls, we evaluated the activity of invertase in the pod racemes of plants in both treatments. The results showed that vacuolar and cytosolic invertase activity did not differ significantly (Figure 4). In contrast, cell wall invertase activity (INVCW) was statistically higher in the walls of small pods with and without water restriction but lower in walls of large pods at both moisture levels (Figure 4). These results suggest that cell wall invertase activity increases in tissues where sink strength is higher, regardless of the plant stress level. Currently, specialists know that cell wall invertase (INVCW) controls growth and development, mainly in reproductive tissues in broad bean (*Vicia faba*) [39], corn (*Zea mays*) [4], and carrot (*Daucus carota*) [43]. The evidence indicates that in these tissues, photoassimilates are unloaded from the phloem and sucrose degradation by cell wall invertase produces hexoses that are incorporated by H+/symport-type transporters (STPs) [44]. However, the presence of six *INVCW* genes and fourteen STPs in *Arabidopsis* has made research difficult due to the redundancy among isoforms [45].

In common bean pods and other legumes, the role of invertases is poorly understood. In asparagus bean (*Vigna unguiculata* ssp. *sesquipedialis*), the cell wall invertase activity in the pod wall has been found to differ between two genotypes; this result indicates that the products of sucrose degradation, glucose, and fructose participate in seed development [46].

The results of the present study indicate that the high activity of INVCW in pod walls of small fruits of plants in both treatments was due to the greater sink strength during seed development (Figure 4).

To determine the number of sequences that encode cell wall invertases in the bean genome, the genome sequence of *P. vulgaris* was downloaded from the free Phytozome V.13 site. After sequence comparison (Table S2), six sequences with a minimum similarity of 78% were identified. With the gene sequences identified in the common bean genome, amino acid sequences were obtained, which were used to carry out the alignments. The analysis showed high similarity among the sequences (Figure 5). However, an intron/exon structure of some fructosyltransferases and invertases includes the presence of a 9-bp mini-exon encoding three amino acids (DNP), which constitutes part of the WMN**DPN**G motif that determines substrate specificity [47]; in the PvINVCW4 sequence, this mini-exon showed an A substitution in the G motif (Figure 5). This mini-exon has been found to show alternative splicing in potato plants under cold stress [48], and a splice variant with different exons and introns has been observed in cotton [40]. Similarly, the results showed other identical RPD and EC motifs in the six sequences identified in beans. Additionally, in the present study, the WECP motif, which has been reported near the terminal carboxyl end [47], was observed in the common bean sequences, with a substitution of C for W. This motif has been identified as the fructofuranoside motif.

The evolutionary distance between them shows that the sequences of PvINVCW3 and PvINVCW5 are distant from those of PvINVCW1, and PvINVCW2 and PvINVCW6 are distant from PvINVCW4 (Figure 6).

For analysis of transcript levels of *PvINVCW* genes, we assessed, by RT–qPCR, the expression of *PvINVCW3* and *PvINVCW4* genes based on the criterion of percentage amino acid similarly ≥ 80% to gene family members of INVCW-like genes (Table S4). The results showed that expression of the *PvINVCW3* gene did not differ significantly between small and long pods, while the levels of *PvINVCW4* gene transcripts were higher in small pods (Figure 7). Recently, Ref. [24] identified five transcription factors (TFs) in reproductive tissues of *Arabidopsis* that differentially regulate the transcriptional activity of two isoforms of cell wall invertase genes, suggesting that TFs participate upstream in different isoforms.

**Figure 8:**
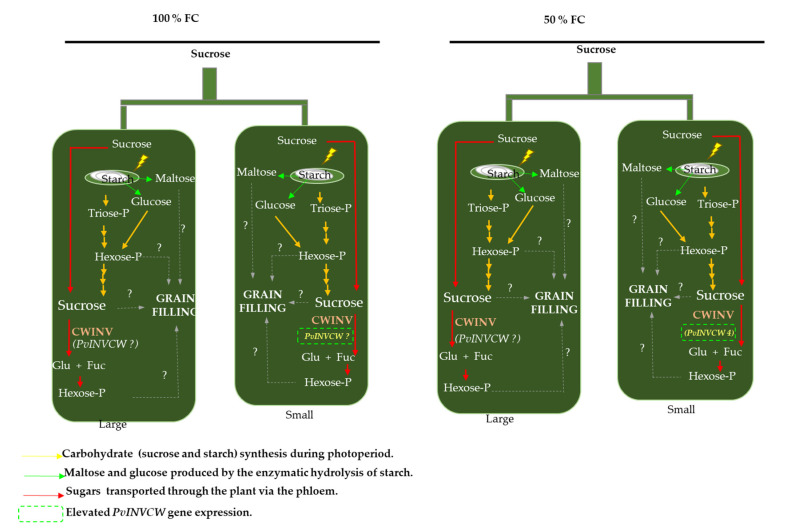
Representative model showing multiple pathways of carbon metabolism during grain filling in racemes at 100% FC and 50% FC. First, the pod wall could perform photosynthesis for starch and sucrose synthesis. Second, products of starch hydrolysis (maltose and hexose) could be used for carbon metabolism in seeds. Third, sucrose could be transported to the peduncle and distributed in large and small pods via funicule to seed. Then, it is degraded by cell wall invertase (INVCW), and hexoses are probably used for grain filling. The activity of INVCW increased in the walls of small pods independent of the water regime, but fructose levels decreased in the walls of large and small pods under water restriction. The mRNA transcript levels of the *PvINVCW4* gene were consistent with high enzymatic activity in small pericarps at 50% FC, but this increase was not mimicked in walls of large pods. These findings suggest that an additional isoform of INVCW could participate in sucrose degradation.

Based on these findings, we conclude that in racemes, multiple pathways of carbon metabolism are affected by the demand in small pods, maintaining the stable morphological and anatomical characteristics under water restriction (Figure 8). Our results pointed to the changes in glucose and fructose that may be associated with cell wall invertase activity (INVCW), and according to the genome reported in Phytozome, six sequences encode INVCW. In this regard, future studies to identify the regulatory factors that can coregulate *CWIN* and additional carbon routes could contribute.

## 4. Materials and Methods

### 4.1. Plant Material and Growth Conditions

Common bean plants of cv. OTI were grown in a tunnel greenhouse of the Colegio de Postgraduados, Campus Montecillo, at Texcoco, Estado de México (19°27′40″ N, 98°54′19″ W and altitude of 2353 m). Plants were distributed in a completely randomized manner and individually maintained in 5-L pots containing 4 kg of agricultural soil at 100% FC until the onset of pod filling at the beginning of the R8 stage. The plants were then separated into two groups: one group was kept at 100% FC (control), and the second group was maintained for 10 days at 50% FC (moisture deficit) (Figure S1). Moisture loss was determined using the gravimetric method by recording the weight of each pot daily at 8:30 a.m. Due to fluctuations in moisture loss during the day, the pots were brought to their respective field capacity (Figure S2). All flowers on each plant were labeled daily and quantified in both treatments, and raceme formation was registered. The racemes at 26–29 days after anthesis (DAA) (R8 stage) were selected for evaluation. The labeled racemes were collected at sunset (6 p.m.), midnight (12 a.m.), dawn (6 a.m.), and noon (12 p.m.) to quantify glucose, fructose, sucrose, and starch concentrations in pod walls (Figure S1).

### 4.2. Phenotyping Analysis

Nine days before collecting pod racemes, lateral RGB (red, green, and blue) images were obtained in one plane of orientation using the Scanalyzer PL phenotyping platform (LemnaTec GmbH, Aachem, Germany). The resolution of the digital camera (Baster AG, Ahrensburg, Germany) was 1628 × 1236 pixels using light in the RGB (400–700 nm) visible spectrum with a pixel size of 4.4 μm × 4.4 μm. The plants were imaged and analyzed with LemnaGrid software, and each image was segmented into green (healthy), yellow (senescent), and brown (necrotic) colors related to the physiology and phenology of the tissues [32].

### 4.3. Anatomical Analysis

Thick sections (0.5 cm) of large and small pod wall, of control (100 % FC) and stressed (50% FC) plants, harvested at 26–29 DAA, were dissected. These were fixed with FAA (formaldehyde, 37% glacial acetic acid, 95% ethanol 95% and distilled water: Ruzin 1999). Then, the sections were dehydrated gradually with alcohol at 30%, 50%, and 70% and with butilic alcohol in a gradient from 10% to 100% in a tissue processor (Leica, TP1020, Deer Park, IL, USA) These samples were included in Paraplast^®^. Ultrathin sections (thickness 12 μm) were obtained with a microtom (Leica, RM2125RT). Deer Park, IL, USA). The ultrathin sections were stained with safranin and fast green and mounted in synthetic resin. Descriptions were visualized with an Olympus BX51 microscope.

### 4.4. Pod and Seed Production

The flowers were labeled and quantified daily in both treatments. The total pod raceme developed during 26–29 DDA was quantified, and the seeds mass produced was determined in an electronic balance (Hongzuan HZ-2003).

### 4.5. Soluble Sugar and Starch Measurements

Soluble sugar and starch were quantified in the pod walls of large and small pods of racemes, harvested after 26–29 DDA, four times in the 24 h cycle. Sucrose, glucose, fructose, and starch were enzymatically quantified following the methods described by [49,50].

### 4.6. Invertase Activity Assay

The invertase activity of cell wall (INVCW), citosolic (CInv), and vacuolar (VInv) were assayed in lyophilized tissue from pod walls of large and small pods harvested at 26–29 DAA. The samples were triturated with a mortar and pestle. For enzyme assays, of triturated samples (50 mg) were homogenized in ice-cold 50 mM HEPES-KOH buffer (pH 8.0), containing 5 mM EDTA, 5 mM MgCl_2_, 1 mM MnCl_2_, 1 mM CaCl_2_, 1 mM DTT, and Coktail of proteases (Sigma-Aldrich, St. Louis, Mo, USA). Then, the samples were vigorously vortexed and centrifuged (10,000× *g*) for 10 min at 4 °C. Assays were performed using the supernatant for soluble invertases, including vacuolar invertases, and in the pellet the acid invertase activity was assayed according to [51].

### 4.7. Identification of PvINVCW Genes in P. vulgaris

#### 4.7.1. Database Search

Gene family members of INVCW-like genes were searched from the most likely sequenced genomes and gene annotations in homologous species: *Zea mays* L. [52] and isoforms of *Oryza sativa* L. [53], *Brachypodium distachyon* [54], *Arabidopsis thaliana* L. [55], *Carica papaya* [56], *Populus trichocarpa* [57], and bacterial *Bacillus subtilis* as the sequence external group (https://www.ncbi.nlm.nih.gov/gene/938745, accessed on 30 January 2022). The sequences were BLASTED by iterative searches at the protein level with high similarity using a threshold >78% against the genome of *P. vulgaris* available Phytozome website version v13. (https://phytozome-next.jgi.doe.gov/info/Pvulgaris_v2_1, accessed on 6 February 2022) [58]. To validate the genes selected, paralogs and orthologs of INVCW from other plant genomes, were searched in GenBank. The exon/intron structure of individual genes was illustrated using the Gene Structure Display Server (GSDS) software [59].

#### 4.7.2. Phylogenetic Analysis

To perform the phylogenetic analysis, we used invertase amino acid sequences of 15 dicots, 25 monocots (Table 1) and selected *P. vulgaris* INVCW entries. Multiple sequence alignments were performed with Clustal X software [60]. Phylogenetic reconstruction was carried out using maximum likelihood (ML) constructed using MEGA X with 1000 according to the amino acid sequence with 1000 bootstrap replicates. The evolution model was selected under the Akaike criterion using the MEGA X program [61]. Additionally, one bacterial sequence was used as an external species.

#### 4.7.3. Bioinformatic Analysis

The molecular weight (MW) and isoelectric point (pI) were calculated using ProtParam (https://web.expasy.org/protparam/, accessed on 10 February 2022) [62]. dbCAN-seq: a database of carbohydrate-active enzyme (CAZyme, http://www.cazy.org/Home.html, accessed on 10 February 2022) sequences and annotations [63]. Carbohydrate-active enzymes (CAZymes) catalyze the assembly and breakdown of glycans and glycoconjugates [64]. SignalP 4.1 predicts the presence and location of signal peptide cleavage sites in amino acid sequences (https://services.healthtech.dtu.dk/service.php?SignalP-4.1, accessed on 11 February 2022).

#### 4.7.4. Reverse Transcription-Quantitative Polymerase Chain Reaction RT–qPCR

Total RNA was isolated from pod sets (large and small pod walls) harvested at 12 p.m. using the TRIzol^®^ reagent (Invitrogen) based on the method of [65] with minor modifications. cDNA templates for qRT–PCR amplification were prepared from pooled RNA from the three individual pod wall of both treatments by using specific primers (Table S1) and SuperScript™ III reverse transcriptase (Invitrogen) according to the manufacturer’s instructions. The PCR cycle was 3 min at 95 °C, followed by 40 cycles of 95 °C for 15 s and 60 °C for 45 s. The specificity of the individual PCR amplification was checked using a heat dissociation protocol from 65 to 95 °C following the final cycle of the PCR. Each reaction contained 20 µg cDNA template obtained from ∼30 µg total RNA, 1× SYBR Green PCR Master Mix (Applied Biosystems, Waltham, MA, USA) and 500 nM forward and reverse primers. Real-time PCR was performed in an ABI PRISM 7500 sequence detection system (Applied Biosystems). Relative transcript abundance was calculated and normalized with respect to actin11 mRNA levels to minimize variation in cDNA template levels and was used as an internal control to normalize gene expression values. The relative expression was calculated based on the increases in pods under humidity restriction with respect to those determined under irrigation. The data shown are the mean values obtained from at least three independent reactions. All calculations and analyses were performed using 7500 Software v2.0.1 (Applied Biosystems, Waltham, MA, USA) and the 2^−ΔΔCt^ method with a relative quantification (RQ) confidence set at 95% [66]. The amplification efficiency (97.4 % to 100 %) for the primer sets was determined by amplification of a cDNA dilution series (1∶5). The specificity of the RT–PCR products was determined by a melting curve analysis with continual fluorescence data acquisition during the 65–95 °C melt.

## Figures and Tables

**Table 1:** Characteristics and properties of INVCWs isoforms in *Phaseolus vulgaris* L.

Name in This Work	Subcellular Location	DNA Length (bp)	mRNA Length (bp)	CDS Length (bp)	Protein Length a.a.	Molecular Weight (Kda)	pI
PvINVCW1	Chr03:44577343..44581434 reverse	4091	2448	1956	652	72.63	6.47
PvINVCW2	Chr10:42023601..42028333 reverse	4732	1823	1689	563	63.51	9.82
PvINVCW3	Chr07:35070399..35075678 forward	5279	1922	1764	588	58.65	9.47
PvINVCW4	Chr01:3435432..3438180 reverse	2748	1859	1686	562	64.12	5.05
PvINVCW5	Chr01:45038437..45040759 forward	2322	1850	1524	508	64.83	8.98
PvINVCW6	Chr05:38694474..38697361 reverse	2887	2007	1725	575	64.65	8.42

## Data Availability

Data are contained within the article.

## Supplementary Materials

The following supporting information can be downloaded at: https://www.mdpi.com/article/10.3390/plants11131622/s1, Figure S1: A representation of the experimental layout and phenological stages of common bean var. OTI plants. VF: vegetative stages and RF: reproductive stages. Plants in R8 were maintained at 100% FC (field capacity) or kept for 10 d at 50% FC. Pod sets were sampled at sunset (6:00 p.m.), midnight (12:00 a.m.), dawn (6:00 a.m.) and noon (12:00 p.m.); Figure S2. Water lost ± SE from substrate per pot registered daily at 8:30 a.m.; water was added to reach 100% FC (blue squares) and 50% FC (red squares). *n* = 10. Figure S3. Schematic representation of the structure of the six cell wall invertases genes identified in *Phaseolus vulgaris* genome. The blue box represent the exons, grey box the untranslated regions (UTRs) and grey lines between boxes represent introns. Table S1. Gene specific primers of INVs used for qPCR amplification; Table S2. List of functionally characterized gene family members of INVCW-like genes searched from the most probable sequenced genomes and gene annotations in homologs species; Table S3. Percentage of similarity obtained by BLAST iterative searches at the protein level against the genome of *Phaseolus vulgaris* available Phytozome website version v12. Table S4. Details of *PvINVCW* genes obtained from the list of functionally characterized gene family members of INVCW-like genes (Table S2) and percentage amino acid similarity >78%.
