# Supplementary material for: Phenotypic, Anatomical, and Diel Variation in Sugar Concentration Linked to Cell Wall Invertases in Common Bean Pod Racemes under Water Restriction
Source: Plants (Basel). 2022 Jun 21;11(13):1622. doi: 10.3390/plants11131622 (PMC9268661; doi:10.3390/plants11131622)
Supplement: Supplementary file 1 [file plants-11-01622-s001.zip › plants-1743004-supplementary.pdf]

Supplementary Material

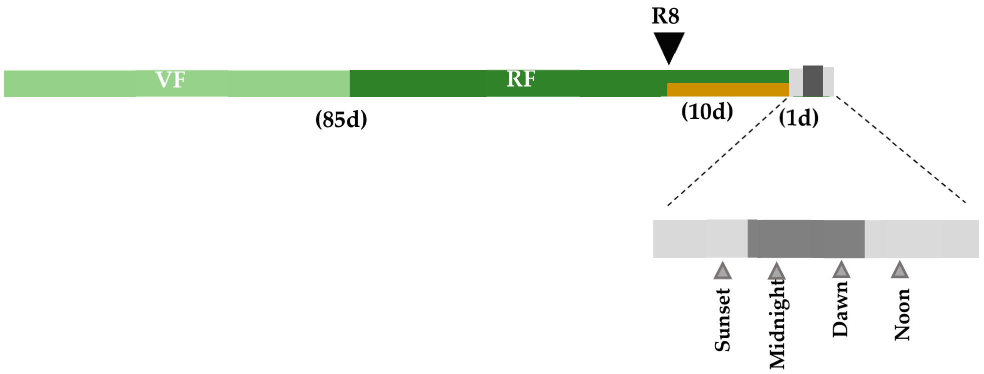

Figure S1. A representation of the experimental layout and phenological stages of common bean var. OTI plants. VF: vegetative stages and RF: reproductive stages. Plants in R8 were maintained at 100% FC (field capacity) or kept for 10 d at 50% FC. Pod sets were sampled at sunset (6:00 pm), midnight (12:00 am), dawn (6:00 am) and noon (12:00 pm).

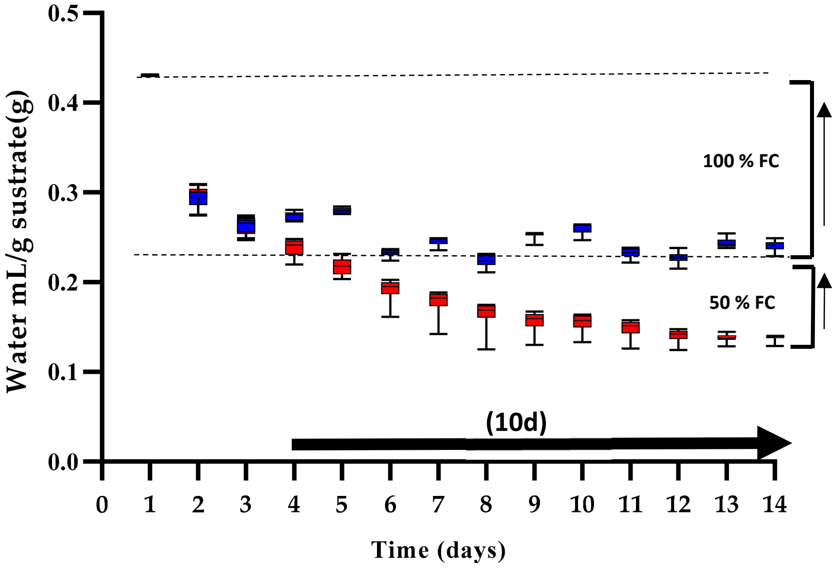

Figure S2. Water lost ± SE from substrate per pot registered daily at 8:30 am; water was added to reach 100% FC (blue squares) and 50% FC (red squares). $n = 10$.

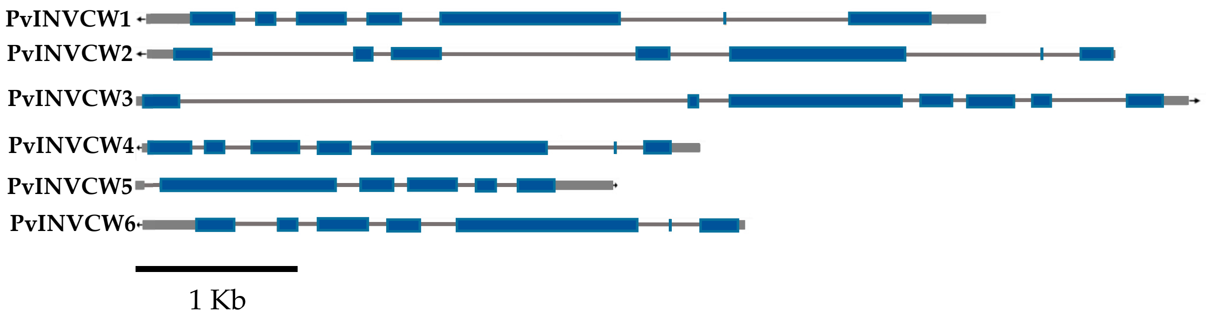

Figure S3. Schematic representation of the structure of the six Cell Wall invertases genes identified in *Phaseolus vulgaris*. The blue box represent the exons, grey box the untranslated regions (UTRs) and grey lines between boxes represent introns.

Table S1. Gene specific primers of INVs used for qPCR amplification

Gen	Forward oligonucleótido 5´- 3´	Reverse oligonucleótido 5´- 3´	Amplicon size (pb)
PvINVCW3	GGACGGACATTGGAGGATATTG	AATGGATCGGGTGCTTGAC	113
PvINVCW4	CAATGTTACGCTGTACCCGAAGA	CGCACTGCCGTTTACTTCTT	111
Act11	TGCATACGTTGGTGATGAGG	AGCCTTGGGGTTAAGAGGAG	

Table S2. List of functionally characterized gene family members of CWINV-like genes searched from the most probable sequenced genomes and gene annotations in homologs species.

Class	Specie	ID	Gene	Family	Chromosome/Scaffold
Monocotyledon	*Brachypodium distachyon*	Bradi1g09500.1	Bradi1g09500	GH32	Bd1
Monocotyledon	*Brachypodium distachyon*	Bradi5g09430.1	Bradi5g09430	GH32	Bd5
Monocotyledon	*Brachypodium distachyon*	Bradi5g09420.1	Bradi5g09420	GH32	Bd5
Monocotyledon	*Brachypodium distachyon*	Bradi3g44990.1	Bradi3g44990	GH32	Bd3
Monocotyledon	*Brachypodium distachyon*	Bradi2g61830.1	Bradi2g61830	GH32	Bd2
Monocotyledon	*Brachypodium distachyon*	Bradi3g46600.1	Bradi3g46600	GH32	Bd3
Monocotyledon	*Brachypodium distachyon*	Bradi4g07847.1	Bradi4g07847	GH32	Bd4
Monocotyledon	*Brachypodium distachyon*	Bradi5g25270.1	Bradi5g25270	GH32	Bd5
Monocotyledon	*Oryza sativa*	LOC_Os03g52560.1	LOC_Os03g52560	GH32	Chr3
Monocotyledon	*Oryza sativa*	LOC_Os04g33720.1	LOC_Os04g33720	GH32	Chr4
Monocotyledon	*Oryza sativa*	LOC_Os04g33740.1	LOC_Os04g33740	GH32	Chr4
Monocotyledon	*Oryza sativa*	LOC_Os02g33110.1	LOC_Os02g33110	GH32	Chr2
Monocotyledon	*Oryza sativa*	LOC_Os01g73580.1	LOC_Os01g73580	GH32	Chr1
Monocotyledon	*Oryza sativa*	LOC_Os04g56930.1	LOC_Os04g56930	GH32	Chr4
Monocotyledon	*Oryza sativa*	LOC_Os04g56920.1	LOC_Os04g56920	GH32	Chr4
Monocotyledon	*Oryza sativa*	LOC_Os09g08120.1	LOC_Os09g08120	GH32	Chr9
Monocotyledon	*Oryza sativa*	LOC_Os09g08072.1	LOC_Os09g08072	GH32	Chr9
Monocotyledon	*Zea mays L*	GRMZM2G139300_T01	GRMZM2G139300	GH32	Chr5
Monocotyledon	*Zea mays L*	GRMZM2G119689_T01	GRMZM2G119689	GH32	Chr2
Monocotyledon	*Zea mays L*	GRMZM2G123633_T01	GRMZM2G123633	GH32	Chr10
Monocotyledon	*Zea mays L*	GRMZM2G119941_T01	GRMZM2G119941	GH32	Chr2
Monocotyledon	*Zea mays L*	GRMZM2G095725_T01	GRMZM2G095725	GH32	Chr10
Monocotyledon	*Zea mays L*	GRMZM2G018692_T01	GRMZM2G018692	GH32	Chr2
Monocotyledon	*Zea mays L*	GRMZM2G018716_T01	GRMZM2G018716	GH32	Chr2
Monocotyledon	*Zea mays L*	GRMZM2G174249_T01	GRMZM2G174249	GH32	Chr3
Dicotyledon	*Arabidopsis thaliana*	AT1G62660.1	AT1G62660	GH32	Chr1
Dicotyledon	*Arabidopsis thaliana*	AT1G12240.1	AT1G12240	GH32	Chr1
Dicotyledon	*Arabidopsis thaliana*	AT5G11920.1	AT5G11920	GH32	Chr5
Dicotyledon	*Arabidopsis thaliana*	AT2G36190.1	AT2G36190	GH32	Chr2
Dicotyledon	*Arabidopsis thaliana*	AT3G52600.1	AT3G52600	GH32	Chr3
Dicotyledon	*Arabidopsis thaliana*	AT3G13790.1	AT3G13790	GH32	Chr3
Dicotyledon	*Populus trichocarpa*	Potri.016G077400.1	Potri.016G077400	GH32	Chr16
Dicotyledon	*Populus trichocarpa*	Potri.016G077500.1	Potri.016G077500	GH32	Chr16
Dicotyledon	*Populus trichocarpa*	Potri.006G210600.1	Potri.006G210600	GH32	Chr6
Dicotyledon	*Populus trichocarpa*	Potri.006G227500.1	Potri.006G227500	GH32	Chr6
Dicotyledon	*Populus trichocarpa*	Potri.006G227400.1	Potri.006G227400	GH32	Chr6
Dicotyledon	*Carica papaya*	evm.TU.supercontig_249.5	evm.model.supercontig_249.5	GH32	supercontig_249
Dicotyledon	*Carica papaya*	evm.TU.supercontig_104.108	evm.model.supercontig_104.108	GH32	supercontig_104

Table S3. Percentage of similarity obtained by BLAST iterative searches at the protein level against the genome of *Phaseolus vulgaris* available Phytozome website version v12.

Species	Percentage amino acid similarity
Brachypodium distachyon	74,7%
Brachypodium distachyon	74,4%
Brachypodium distachyon	76,3%
Brachypodium distachyon	76,3%
Brachypodium distachyon	68,2%
Brachypodium distachyon	67,3%
Brachypodium distachyon	65,7%
Brachypodium distachyon	66%
Oryza sativa	70,3%
Oryza sativa	73,4%
Oryza sativa	73,6%
Oryza sativa	74,9%
Oryza sativa	68,9%
Oryza sativa	68,5%
Oryza sativa	67,2%
Oryza sativa	67,5%
Oryza sativa	69,2%
Zea mays L	73,4%
Zea mays L	74,9%
Zea mays L	72,6%
Zea mays L	67,6%
Zea mays L	73%
Zea mays L	65,8%
Zea mays L	63,7%
Zea mays L	65,2%
Arabidopsis thaliana	82,5%
Arabidopsis thaliana	82,6%
Arabidopsis thaliana	73,9%
Arabidopsis thaliana	79,5%
Arabidopsis thaliana	78,6%
Arabidopsis thaliana	78,4%
Populus trichocarpa	82,4%
Populus trichocarpa	80,4%
Populus trichocarpa	82,7%
Populus trichocarpa	78,7%
Populus trichocarpa	78,5%
Carica papaya	73,3%
Carica papaya	79,3%

Table S4. Details of *PvINVCW* genes obtained from the list of functionally characterized Gene family members of CWINV-like genes (Table S2) and percentage of amino acid similarity > 78 %.

Name in this work	ID *Phaseolus vulgaris*	Gen ID	Percentage of amino acid similarity	Reference
PvINVCW1	Phvul.003G217900.1	*AT1G12240*	82.60%	(Qi , *et al.*, 2007)
PvINVCW2	Phvul.010G137900.1	*AT3G13790*	78.40%	(Qi , *et al.*, 2007)
PvINVCW3	Phvul.007G227200.2	*Potri.016G077500.1*	80.40%	(Chen *et al.*, 2015)
PvINVCW4	Phvul.001G191600.2	*Potri.006G210600.1*	82,7%	(Chen *et al.*, 2015)
PvINVCW5	Phvul.001G036700.1	*Potri.006G227500.1*	78,7%	(Chen *et al.*, 2015)
PvINVCW6	Phvul.005G158500.1	*Potri.006G227400.1*	78.50%	(Chen *et al.*, 2015)
